# 2-(4-Fluoro­phen­yl)-5-iodo-7-methyl-3-methyl­sulfinyl-1-benzofuran

**DOI:** 10.1107/S1600536810022397

**Published:** 2010-06-16

**Authors:** Hong Dae Choi, Pil Ja Seo, Byeng Wha Son, Uk Lee

**Affiliations:** a Department of Chemistry, Dongeui University, San 24 Kaya-dong Busanjin-gu, Busan 614-714, Republic of Korea; b Department of Chemistry, Pukyong National University, 599-1 Daeyeon 3-dong, Nam-gu, Busan 608-737, Republic of Korea

## Abstract

In the title compound, C_16_H_12_FIO_2_S, the O atom and the methyl group of the methyl­sulfinyl substituent lie on opposite sides of the plane through the benzofuran fragment. The 4-fluoro­phenyl ring is rotated slightly out of the benzofuran plane, as indicated by the dihedral angle of 7.43 (6)°. In the crystal structure, pairs of short I⋯O [3.074 (2) Å] contacts link the mol­ecules into centrosymmetric dimers. These dimers are further linked *via* aromatic π–π inter­actions between the benzene and the 4-fluoro­phenyl rings of neighbouring mol­ecules [centroid–centroid distance = 3.617 (3) Å].

## Related literature

For the crystal structures of similar 3-ethyl­sulfinyl-2-(4-fluoro­phen­yl)-5-halo-7-methyl-1-benzofuran derivatives, see: Choi *et al.* (2010**a* ▶,b* ▶). For the pharmacological activity of benzofuran compounds, see: Aslam *et al.* (2006 ▶); Galal *et al.* (2009 ▶); Khan *et al.* (2005 ▶). For natural products with benzofuran rings, see: Akgul & Anil (2003 ▶); Soekamto *et al.* (2003 ▶). For a review of halogen bonding, see: Politzer *et al.* (2007 ▶).
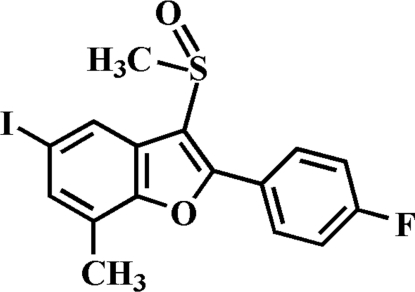


## Experimental

### 

#### Crystal data

C_16_H_12_FIO_2_S
*M* *_r_* = 414.22
Triclinic, 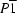

*a* = 7.3828 (2) Å
*b* = 9.8680 (3) Å
*c* = 11.0670 (4) Å
α = 74.979 (1)°
β = 83.511 (1)°
γ = 70.011 (1)°
*V* = 731.54 (4) Å^3^
*Z* = 2
Mo *K*α radiation
μ = 2.34 mm^−1^
*T* = 174 K
0.35 × 0.23 × 0.16 mm

#### Data collection

Bruker SMART APEXII CCD diffractometer
Absorption correction: multi-scan (*SADABS*; Bruker, 2009 ▶) *T*_min_ = 0.494, *T*_max_ = 0.706
12823 measured reflections
3364 independent reflections
3283 reflections with *I* > 2σ(*I*)
*R*_int_ = 0.026

#### Refinement

*R*[*F* ^2^ > 2σ(*F* ^2^)] = 0.021
*wR*(*F* ^2^) = 0.056
*S* = 1.16
3364 reflections
192 parameters
H-atom parameters constrained
Δρ_max_ = 0.38 e Å^−3^
Δρ_min_ = −0.90 e Å^−3^

### 

Data collection: *APEX2* (Bruker, 2009 ▶); cell refinement: *SAINT* (Bruker, 2009 ▶); data reduction: *SAINT*; program(s) used to solve structure: *SHELXS97* (Sheldrick, 2008 ▶); program(s) used to refine structure: *SHELXL97* (Sheldrick, 2008 ▶); molecular graphics: *ORTEP-3* (Farrugia, 1997 ▶) and *DIAMOND* (Brandenburg, 1998 ▶); software used to prepare material for publication: *SHELXL97*.

## Supplementary Material

Crystal structure: contains datablocks global, I. DOI: 10.1107/S1600536810022397/vm2030sup1.cif

Structure factors: contains datablocks I. DOI: 10.1107/S1600536810022397/vm2030Isup2.hkl

Additional supplementary materials:  crystallographic information; 3D view; checkCIF report

## Figures and Tables

**Fig. 1.**
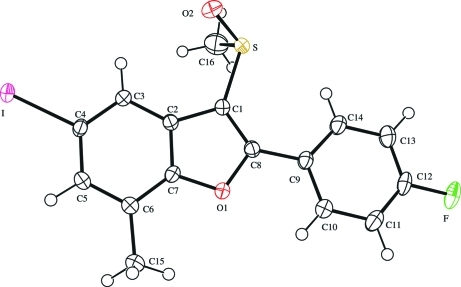
The molecular structure of the title compound with the atom numbering scheme. Displacement ellipsoids are drawn at the 50% probability level. H atoms are presented as a small spheres of arbitrary radius.

**Fig. 2.**
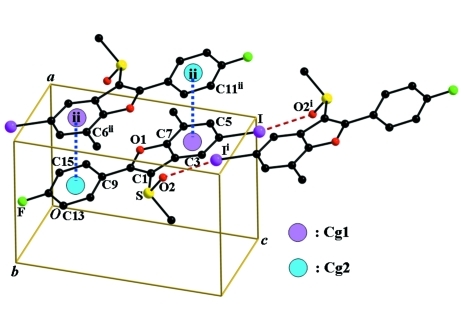
I···O and π–π interactions (dotted lines) in the crystal structure of the title compound. Cg denotes the ring centroids. [Symmetry codes: (i) - x + 2, - y + 1, - z + 2; (ii) - x + 2, - y + 1, - z + 1.]

## supplementary crystallographic information

### Comment

The compounds involving a benzofuran moiety show interesting pharmacological properties such as antifungal (Aslam *et al.*, 2006), antitumor and antiviral (Galal *et al.*., 2009), antimicrobial (Khan *et al.*., 2005) activities. These compounds occur in nature (Akgul & Anil, 2003; Soekamto *et al.*., 2003). As a part of our ongoing studies of the effect of side chain substituents on the solid state structures of 3-ethylsulfinyl-2-(4-fluorophenyl)-5-halo-7-methyl-1-benzofuran analogues (Choi *et al.*., 2010*a, b*), we report the crystal structure of the title compound (Fig. 1).

The benzofuran unit is essentially planar, with a mean deviation of 0.007 (1) Å from the least-squares plane defined by the nine constituent atoms. The dihedral angle formed by the benzofuran plane and the 4-fluorophenyl ring is 7.43 (6)°. The crystal packing (Fig. 2) is stabilized by I···O halogen bondings between the iodine and the oxygen of the S═O unit [I···O2^i^ = 3.074 (2) Å; C4—I···O2^i^ = 167.82 (6)°] (Politzer *et al.*, 2007). The molecular packing is further stabilized by aromatic π–π interactions between the benzene and the 4-fluorophenyl rings of adjacent molecules, with a Cg1···Cg2^ii^ distance of 3.617 (3) Å (Cg1 and Cg2 are the centroids of the the C2–C7 benzene ring and the C9–C14 4-fluorophenyl ring, respectively).

### Experimental

77% 3-chloroperoxybenzoic acid (269 mg, 1.2 mmol) was added in small portions to a stirred solution of 2-(4-fluorophenyl)-5-iodo-7-methyl-3-methylsulfanyl-1-benzofuran (438 mg, 1.1 mmol) in dichloromethane (35 mL) at 273 K. After being stirred at room temperature for 4hr, the mixture was washed with saturated sodium bicarbonate solution and the organic layer was separated, dried over magnesium sulfate, filtered and concentrated at reduced pressure. The residue was purified by column chromatography (hexane-ethyl acetate, 1:1 v/v) to afford the title compound as a colorless solid [yield 77%, m.p. 502–503 K; *R*_f_ = 0.57 (hexane–ethyl acetate, 1:1 v/v)]. Single crystals suitable for X-ray diffraction were prepared by slow evaporation of a solution of the title compound in tetrahydrofuran at room temperature.

### Refinement

All H atoms were positioned geometrically and refined using a riding model, with C—H = 0.95 Å for aryl and 0.98 Å for methyl H atoms. *U*_iso_(H) = 1.2*U*_eq_(C) for aryl and 1.5*U*_eq_(C) for methyl H atoms.

### Crystal data

**Table:**

C_16_H_12_FIO_2_S	*Z* = 2
*M**_r_* = 414.22	*F*(000) = 404
Triclinic, *P*1	*D*_x_ = 1.880 Mg m^−^^3^
Hall symbol: -P 1	Mo *K*α radiation, λ = 0.71073 Å
*a* = 7.3828 (2) Å	Cell parameters from 9921 reflections
*b* = 9.8680 (3) Å	θ = 2.3–27.5°
*c* = 11.0670 (4) Å	µ = 2.34 mm^−^^1^
α = 74.979 (1)°	*T* = 174 K
β = 83.511 (1)°	Block, colourless
γ = 70.011 (1)°	0.35 × 0.23 × 0.16 mm
*V* = 731.54 (4) Å^3^	

### Data collection

**Table:**

Bruker SMART APEXII CCD diffractometer	3364 independent reflections
Radiation source: rotating anode	3283 reflections with *I* > 2σ(*I*)
graphite multilayer	*R*_int_ = 0.026
Detector resolution: 10.0 pixels mm^-1^	θ_max_ = 27.5°, θ_min_ = 1.9°
φ and ω scans	*h* = −9→9
Absorption correction: multi-scan (*SADABS*; Bruker, 2009)	*k* = −12→12
*T*_min_ = 0.494, *T*_max_ = 0.706	*l* = −14→14
12823 measured reflections	

### Refinement

**Table:**

Refinement on *F*^2^	Primary atom site location: structure-invariant direct methods
Least-squares matrix: full	Secondary atom site location: difference Fourier map
*R*[*F*^2^ > 2σ(*F*^2^)] = 0.021	Hydrogen site location: difference Fourier map
*wR*(*F*^2^) = 0.056	H-atom parameters constrained
*S* = 1.16	*w* = 1/[σ^2^(*F*_o_^2^) + (0.0298*P*)^2^ + 0.3131*P*] where *P* = (*F*_o_^2^ + 2*F*_c_^2^)/3
3364 reflections	(Δ/σ)_max_ < 0.001
192 parameters	Δρ_max_ = 0.38 e Å^−^^3^
0 restraints	Δρ_min_ = −0.90 e Å^−^^3^

### Special details

**Table:**

Geometry. All esds (except the esd in the dihedral angle between two l.s. planes) are estimated using the full covariance matrix. The cell esds are taken into account individually in the estimation of esds in distances, angles and torsion angles; correlations between esds in cell parameters are only used when they are defined by crystal symmetry. An approximate (isotropic) treatment of cell esds is used for estimating esds involving l.s. planes.
Refinement. Refinement of F^2^ against ALL reflections. The weighted R-factor wR and goodness of fit S are based on F^2^, conventional R-factors R are based on F, with F set to zero for negative F^2^. The threshold expression of F^2^ > 2sigma(F^2^) is used only for calculating R-factors(gt) etc. and is not relevant to the choice of reflections for refinement. R-factors based on F^2^ are statistically about twice as large as those based on F, and R- factors based on ALL data will be even larger.

### Fractional atomic coordinates and isotropic or equivalent isotropic displacement parameters (Å^2^)

**Table:**

	*x*	*y*	*z*	*U*_iso_*/*U*_eq_	
I	1.016803 (18)	0.251259 (13)	1.060638 (11)	0.02458 (6)	
S	0.64528 (8)	0.79666 (5)	0.61575 (5)	0.02839 (12)	
F	0.4311 (2)	0.84290 (18)	0.00720 (13)	0.0412 (3)	
O1	0.7930 (2)	0.41864 (14)	0.50775 (13)	0.0214 (3)	
O2	0.7975 (3)	0.80561 (19)	0.68819 (16)	0.0387 (4)	
C1	0.7172 (3)	0.6143 (2)	0.59489 (18)	0.0211 (4)	
C2	0.8070 (3)	0.4821 (2)	0.68908 (18)	0.0196 (3)	
C3	0.8529 (3)	0.4514 (2)	0.81468 (18)	0.0214 (4)	
H3	0.8251	0.5280	0.8580	0.026*	
C4	0.9409 (3)	0.3037 (2)	0.87281 (18)	0.0211 (4)	
C5	0.9866 (3)	0.1884 (2)	0.81050 (19)	0.0229 (4)	
H5	1.0491	0.0892	0.8542	0.027*	
C6	0.9414 (3)	0.2179 (2)	0.68520 (19)	0.0223 (4)	
C7	0.8514 (3)	0.3659 (2)	0.62985 (17)	0.0195 (3)	
C8	0.7117 (3)	0.5708 (2)	0.48766 (18)	0.0200 (4)	
C9	0.6396 (3)	0.6448 (2)	0.36167 (18)	0.0208 (4)	
C10	0.6785 (3)	0.5633 (2)	0.27040 (19)	0.0258 (4)	
H10	0.7546	0.4614	0.2903	0.031*	
C11	0.6073 (3)	0.6292 (3)	0.1511 (2)	0.0304 (4)	
H11	0.6311	0.5732	0.0896	0.036*	
C12	0.5010 (3)	0.7781 (3)	0.12384 (19)	0.0283 (4)	
C13	0.4618 (3)	0.8629 (2)	0.2103 (2)	0.0312 (4)	
H13	0.3894	0.9656	0.1885	0.037*	
C14	0.5304 (3)	0.7952 (2)	0.3300 (2)	0.0280 (4)	
H14	0.5027	0.8518	0.3914	0.034*	
C15	0.9850 (4)	0.0985 (2)	0.6148 (2)	0.0320 (5)	
H15A	0.8996	0.1337	0.5436	0.048*	
H15B	0.9644	0.0098	0.6706	0.048*	
H15C	1.1195	0.0741	0.5844	0.048*	
C16	0.4459 (4)	0.7855 (3)	0.7220 (3)	0.0430 (6)	
H16A	0.4901	0.7012	0.7938	0.065*	
H16B	0.3466	0.7719	0.6791	0.065*	
H16C	0.3919	0.8774	0.7511	0.065*	

### Atomic displacement parameters (Å^2^)

**Table:**

	*U*^11^	*U*^22^	*U*^33^	*U*^12^	*U*^13^	*U*^23^
I	0.03263 (9)	0.02220 (8)	0.01678 (8)	−0.00764 (6)	−0.00759 (5)	0.00021 (5)
S	0.0458 (3)	0.0165 (2)	0.0221 (2)	−0.0078 (2)	−0.0102 (2)	−0.00249 (18)
F	0.0468 (8)	0.0532 (9)	0.0180 (6)	−0.0127 (7)	−0.0132 (6)	0.0018 (6)
O1	0.0266 (7)	0.0186 (6)	0.0172 (7)	−0.0047 (5)	−0.0041 (5)	−0.0036 (5)
O2	0.0602 (11)	0.0337 (8)	0.0308 (9)	−0.0224 (8)	−0.0143 (8)	−0.0073 (7)
C1	0.0265 (9)	0.0177 (8)	0.0190 (9)	−0.0068 (7)	−0.0054 (7)	−0.0025 (7)
C2	0.0219 (9)	0.0174 (8)	0.0195 (9)	−0.0068 (7)	−0.0036 (7)	−0.0023 (7)
C3	0.0263 (9)	0.0197 (8)	0.0194 (9)	−0.0080 (7)	−0.0054 (7)	−0.0040 (7)
C4	0.0245 (9)	0.0230 (9)	0.0161 (9)	−0.0091 (7)	−0.0052 (7)	−0.0015 (7)
C5	0.0260 (9)	0.0166 (8)	0.0235 (10)	−0.0049 (7)	−0.0039 (7)	−0.0016 (7)
C6	0.0253 (9)	0.0188 (8)	0.0219 (9)	−0.0061 (7)	−0.0022 (7)	−0.0041 (7)
C7	0.0208 (8)	0.0219 (9)	0.0163 (9)	−0.0068 (7)	−0.0031 (7)	−0.0043 (7)
C8	0.0207 (8)	0.0185 (8)	0.0200 (9)	−0.0062 (7)	−0.0026 (7)	−0.0028 (7)
C9	0.0211 (9)	0.0242 (9)	0.0175 (9)	−0.0090 (7)	−0.0034 (7)	−0.0023 (7)
C10	0.0303 (10)	0.0258 (9)	0.0209 (10)	−0.0086 (8)	−0.0026 (8)	−0.0048 (8)
C11	0.0360 (11)	0.0380 (12)	0.0188 (10)	−0.0132 (9)	−0.0017 (8)	−0.0077 (9)
C12	0.0282 (10)	0.0394 (12)	0.0154 (9)	−0.0127 (9)	−0.0067 (8)	0.0016 (8)
C13	0.0327 (11)	0.0285 (10)	0.0261 (11)	−0.0045 (8)	−0.0101 (8)	0.0008 (8)
C14	0.0328 (11)	0.0273 (10)	0.0211 (10)	−0.0050 (8)	−0.0081 (8)	−0.0046 (8)
C15	0.0447 (13)	0.0198 (9)	0.0267 (11)	−0.0024 (8)	−0.0047 (9)	−0.0070 (8)
C16	0.0432 (14)	0.0335 (12)	0.0469 (16)	−0.0009 (10)	0.0028 (11)	−0.0175 (11)

### Geometric parameters (Å, °)

**Table:**

I—O2^i^	3.074 (2)	C6—C15	1.503 (3)
I—C4	2.100 (2)	C8—C9	1.461 (3)
S—O2	1.490 (2)	C9—C10	1.396 (3)
S—C1	1.763 (2)	C9—C14	1.396 (3)
S—C16	1.793 (3)	C10—C11	1.386 (3)
F—C12	1.356 (2)	C10—H10	0.9500
O1—C7	1.377 (2)	C11—C12	1.379 (3)
O1—C8	1.380 (2)	C11—H11	0.9500
C1—C8	1.371 (3)	C12—C13	1.373 (3)
C1—C2	1.445 (3)	C13—C14	1.386 (3)
C2—C7	1.391 (2)	C13—H13	0.9500
C2—C3	1.399 (3)	C14—H14	0.9500
C3—C4	1.386 (3)	C15—H15A	0.9800
C3—H3	0.9500	C15—H15B	0.9800
C4—C5	1.405 (3)	C15—H15C	0.9800
C5—C6	1.396 (3)	C16—H16A	0.9800
C5—H5	0.9500	C16—H16B	0.9800
C6—C7	1.384 (3)	C16—H16C	0.9800
			
C4—I—O2^i^	167.82 (6)	C10—C9—C8	119.38 (18)
O2—S—C1	107.34 (10)	C14—C9—C8	121.85 (18)
O2—S—C16	107.16 (12)	C11—C10—C9	120.8 (2)
C1—S—C16	97.31 (11)	C11—C10—H10	119.6
C7—O1—C8	107.11 (14)	C9—C10—H10	119.6
C8—C1—C2	107.37 (16)	C12—C11—C10	118.5 (2)
C8—C1—S	127.77 (15)	C12—C11—H11	120.8
C2—C1—S	124.74 (14)	C10—C11—H11	120.8
C7—C2—C3	119.39 (17)	F—C12—C13	118.8 (2)
C7—C2—C1	105.13 (16)	F—C12—C11	118.55 (19)
C3—C2—C1	135.48 (17)	C13—C12—C11	122.6 (2)
C4—C3—C2	116.70 (17)	C12—C13—C14	118.4 (2)
C4—C3—H3	121.7	C12—C13—H13	120.8
C2—C3—H3	121.7	C14—C13—H13	120.8
C3—C4—C5	122.87 (18)	C13—C14—C9	120.9 (2)
C3—C4—I	118.39 (14)	C13—C14—H14	119.5
C5—C4—I	118.71 (14)	C9—C14—H14	119.5
C6—C5—C4	120.84 (17)	C6—C15—H15A	109.5
C6—C5—H5	119.6	C6—C15—H15B	109.5
C4—C5—H5	119.6	H15A—C15—H15B	109.5
C7—C6—C5	115.12 (17)	C6—C15—H15C	109.5
C7—C6—C15	121.98 (18)	H15A—C15—H15C	109.5
C5—C6—C15	122.90 (18)	H15B—C15—H15C	109.5
O1—C7—C6	124.45 (16)	S—C16—H16A	109.5
O1—C7—C2	110.49 (16)	S—C16—H16B	109.5
C6—C7—C2	125.07 (18)	H16A—C16—H16B	109.5
C1—C8—O1	109.90 (16)	S—C16—H16C	109.5
C1—C8—C9	135.88 (17)	H16A—C16—H16C	109.5
O1—C8—C9	114.21 (16)	H16B—C16—H16C	109.5
C10—C9—C14	118.77 (18)		
			
O2—S—C1—C8	−135.42 (18)	C1—C2—C7—O1	−0.6 (2)
C16—S—C1—C8	114.0 (2)	C3—C2—C7—C6	−0.6 (3)
O2—S—C1—C2	39.9 (2)	C1—C2—C7—C6	179.44 (18)
C16—S—C1—C2	−70.7 (2)	C2—C1—C8—O1	−0.1 (2)
C8—C1—C2—C7	0.5 (2)	S—C1—C8—O1	175.88 (14)
S—C1—C2—C7	−175.69 (14)	C2—C1—C8—C9	178.7 (2)
C8—C1—C2—C3	−179.5 (2)	S—C1—C8—C9	−5.3 (3)
S—C1—C2—C3	4.4 (3)	C7—O1—C8—C1	−0.3 (2)
C7—C2—C3—C4	−0.2 (3)	C7—O1—C8—C9	−179.40 (15)
C1—C2—C3—C4	179.7 (2)	C1—C8—C9—C10	173.3 (2)
C2—C3—C4—C5	1.1 (3)	O1—C8—C9—C10	−7.9 (2)
C2—C3—C4—I	179.11 (13)	C1—C8—C9—C14	−7.5 (3)
C3—C4—C5—C6	−1.2 (3)	O1—C8—C9—C14	171.28 (17)
I—C4—C5—C6	−179.17 (15)	C14—C9—C10—C11	−1.2 (3)
C4—C5—C6—C7	0.3 (3)	C8—C9—C10—C11	178.05 (19)
C4—C5—C6—C15	−179.2 (2)	C9—C10—C11—C12	1.5 (3)
C8—O1—C7—C6	−179.50 (18)	C10—C11—C12—F	−180.0 (2)
C8—O1—C7—C2	0.6 (2)	C10—C11—C12—C13	−0.6 (3)
C5—C6—C7—O1	−179.32 (17)	F—C12—C13—C14	178.7 (2)
C15—C6—C7—O1	0.2 (3)	C11—C12—C13—C14	−0.7 (3)
C5—C6—C7—C2	0.6 (3)	C12—C13—C14—C9	1.1 (3)
C15—C6—C7—C2	−179.88 (19)	C10—C9—C14—C13	−0.2 (3)
C3—C2—C7—O1	179.29 (16)	C8—C9—C14—C13	−179.36 (19)

Symmetry codes: (i) −*x*+2, −*y*+1, −*z*+2.

## References

[bb1] Akgul, Y. Y. & Anil, H. (2003). *Phytochemistry*, **63**, 939–943. 10.1016/s0031-9422(03)00357-1 12895543

[bb2] Aslam, S. N., Stevenson, P. C., Phythian, S. J., Veitch, N. C. & Hall, D. R. (2006). *Tetrahedron*, **62**, 4214–4226.

[bb3] Brandenburg, K. (1998). *DIAMOND* Crystal Impact GbR, Bonn, Germany.

[bb4] Bruker (2009). *SADABS* *APEX2* and *SAINT* Bruker AXS Inc., Madison, Wisconsin, USA.

[bb5] Choi, H. D., Seo, P. J., Son, B. W. & Lee, U. (2010*a*). *Acta Cryst.* E**66**, o629. 10.1107/S1600536810005581 PMC2983596 21580386

[bb6] Choi, H. D., Seo, P. J., Son, B. W. & Lee, U. (2010*b*). *Acta Cryst.* E**66**, o886. 10.1107/S1600536810008627 PMC2983861 21580704

[bb7] Farrugia, L. J. (1997). *J. Appl. Cryst.* **30**, 565.

[bb8] Galal, S. A., Abd El-All, A. S., Abdallah, M. M. & El-Diwani, H. I. (2009). *Bioorg. Med. Chem. Lett* **19**, 2420–2428. 10.1016/j.bmcl.2009.03.069 19345581

[bb9] Khan, M. W., Alam, M. J., Rashid, M. A. & Chowdhury, R. (2005). *Bioorg. Med. Chem* **13**, 4796–4805. 10.1016/j.bmc.2005.05.009 15964760

[bb10] Politzer, P., Lane, P., Concha, M. C., Ma, Y. & Murray, J. S. (2007). *J. Mol. Model* **13**, 305–311. 10.1007/s00894-006-0154-7 17013631

[bb11] Sheldrick, G. M. (2008). *Acta Cryst.* A**64**, 112–122. 10.1107/S0108767307043930 18156677

[bb12] Soekamto, N. H., Achmad, S. A., Ghisalberti, E. L., Hakim, E. H. & Syah, Y. M. (2003). *Phytochemistry*, **64**, 831–834. 10.1016/j.phytochem.2003.08.009 14559276

